# Respiratory sinus arrhythmia reactivity of internet addiction abusers in negative and positive emotional states using film clips stimulation

**DOI:** 10.1186/s12938-016-0201-2

**Published:** 2016-07-04

**Authors:** Dai-Ling Hsieh, Tzu-Chien Hsiao

**Affiliations:** Institute of Computer Science and Engineering, National Chiao Tung University, No. 1001 University Road, Hsinchu, 300 Taiwan; Biomedical Electronics Translational Research Center and Biomimetic Systems Research Center, National Chiao Tung University, No. 1001 University Road, Hsinchu, 300 Taiwan; Institute of Biomedical Engineering, National Chiao Tung University, No. 1001 University Road, Hsinchu, 300 Taiwan; Department of Computer Science, National Chiao Tung University, No. 1001 University Road, Hsinchu, 300 Taiwan

**Keywords:** Internet addiction, Emotion, Autonomic nervous system, Respiratory sinus arrhythmia, Vagus nerve regulation, Linear model

## Abstract

**Background and aims:**

People with internet addiction (IA) suffer from mental, physical, social, and occupational problems. IA includes psychological and physiological syndromes, and among the syndromes, emotion was suggested important mental and physiological expressions of IA. However, few physiologically emotional characters of IA were investigated. Autonomic nervous system (ANS) activity was a good link between IA and emotion, and respiratory sinus arrhythmia (RSA) gained from ANS was hypothesized related to IA.

**Methods:**

An emotional induction experiment using negative and positive emotional films was conducted to validate the hypotheses. Thirty-four participants recruited from college were classified into high-risk IA group (HIA) and low-risk IA group (LIA). The respiratory signals, ECG signals, and self-assessed emotional intensity were acquired. The relationship and difference between IA and RSA was tested using descriptive statistics and inferential statistics.

**Results:**

The RSA values of HIA were lower than those of LIA both before and after the induction of positive and negative emotions. When participants experienced a negative emotion (anger or fear), their RSA values declined; the decline for HIA was greater than that for LIA. The RSA values of HIA participants before induction of fear, happiness, or surprise, statistically significantly differed from that after induction of those emotions, with *p* values of 0.007, 0.04 and 0.01 respectively. The difference between the changes in RSA values upon the induction of surprise of HIA and LIA was statistically significant difference (*p* = 0.03). The interaction between two IA groups among emotional induction states was statistically significant difference.

**Conclusions:**

RSA value here was the main variable that reflected ANS activity, and especially vagus nerve regulation. The results revealed that the changes in RSA values were biologically significantly different between HIA and LIA, especially when sadness, happiness, or surprise was induced. HIA people exhibited stronger RSA reactivity following negative emotion than LIA people, but the RSA reactivity following positive emotion was weaker. This study provides more physiological information about IA and assists further investigation on the regulation of the ANS for IA abusers. The results will benefit the further application, early detection, therapy, and even early prevention.

*Clinical trial registration details* This study was approved by the Institution Review Board of the National Taiwan University Hospital, Hsinchu Branch (Hsinchu, Taiwan), under the research project: A study of interactions between cognition, emotion and physiology (contract no.100IRB-32)

## Background

The population of internet users has reached almost two and a half billion [1], and the internet is now a part of everyday life. Some users, however, have internet addiction (IA), and suffer from related mental, physical, social, and occupational problems [2]. Some widely used definitions of IA include excessive internet use, withdrawal, tolerance, and negative consequences. For example, people with IA usually used computer or internet over 10 hours a day, 6 days a week [3]. The negative consequences were arguments, poor performance of school or work, familial problems, financial difficulty, social isolation and fatigue [1, 2]. The prevalence rates investigated by Norway and the US were 0.7 % and 1 %, respectively. For adolescents, in European and Asian, the prevalence rates ranged from 1.0 % to 9.0 % and from 2.0 % to 18.0 %, respectively [1]. In recently study, the prevalence rates in Hong Kong adolescents ranged from 17 % to 26.8 % during the high school years [4]. In Taiwan, approximately 20 % of adolescent and early adult students were classified as IA [5].

IA includes psychological and physiological syndromes, such as emotional and social withdrawal from real relationship, mood-altering use of the internet, guilt, craving, fatigue, and insomnia [2, 6–10]. Young proposed that emotion can be used to assess IA [2]. Oktan found that emotion management skills were meaningful predictors of IA [11]. People with IA were reported bad scores on emotional intelligence tests [12, 13]. Excessive internet users were indicated poorer intimacy, and worse expressions of positive and negative emotions than others [14]. The withdrawal symptoms of internet gaming disorder (IGD) may indicate an immediate emotional reaction [15]. Among the syndromes, emotion was suggested important mental and physiological expression of IA. The mental expressions of IA were craving, feelings of excitement, euphoria, feeling of security or calm, of self-worth or accomplishment, of power and control, or intimacy or belonging [1, 2], and the physiological expressions were observed from autonomic nervous system (ANS) activity [16].

The physiological characters of emotion are considered to provide objective information and some cannot be controlled consciously, such as ANS activities [17]. The ANS contains sympathetic and parasympathetic nervous systems, which has antagonistic effects. Several ANS responses that are related to emotion are cardiovascular responses, electrodermal responses, and respiratory responses. Some studies investigated the autonomic nervous responses related to high-risk (HIA) and low-risk IA (LIA) abusers. HIA abusers showed weaker peripheral temperature, stronger respiratory response, increased blood volume pulse, and decreased skin conductance than LIA abusers. The sympathetic nervous system of HIA abusers was heavily activated, and in the meantime, parasympathetic responses was activated by skin conductance [16]. Heart rate variability (HRV) is another index of ANS activity. The high frequency (HF) percentage of IA abusers was lower than that of non-IA abusers, but the low frequency (LF) percentage was higher. IA abusers presented higher sympathetic activity and lower parasympathetic activity [18].

However, the physiologically emotional characters of IA were rarely investigated. Base on the literature studies, ANS activity was a good link between IA and emotion. It was noticed that cardiac response and respiratory response importantly affect regulatory functions, and may also be a regulation means to IA. Hence, the related response was worthy to be studied. Respiratory sinus arrhythmia (RSA), the activity of the vagus nerve, is rhythmic fluctuations in the heart rate that are associated with respiration. According to the polyvagal theory, nucleus ambigus fibers support complex emotion responses and social behavior [19, 20]. Both polyvagal theory [19] and a neurovisceral integration model [21] implied that common neural pathways existed in underlying autonomic control and self-regulation. The RSA value reflects the autonomic control activity, and the difference RSA value associated with induction of emotions indicates the changing states of RSA, parasympathetic activity, following emotion stimulations. Therefore, RSA can be utilized to investigate the relationship between emotions and the activity of the vagus nerve by noninvasive means [9]. People with a higher resting RSA level tend to express more positive emotions than negative emotions, and to suppress negative emotional expressions, including facial expressions [22, 23].

It was hypothesized that RSA related to IA. Further, the RSA level between HIA abusers and LIA abusers was hypothesized different in both negative and positive emotional states, and the RSA value of HIA abusers was lower than LIA abusers. An emotional induction experiment using negative and positive emotional films was conducted to validate the hypotheses. The relationship and difference between IA and RSA were tested, and the regulation of the parasympathetic activity in emotional states was discussed.

IA is a fast growing and unavoidable problem around the world and much attention has been paid in recent years. The important diagnostic and statistical manual for mental disorders (DSM-5) reports that IGD/IA should be further studied; more relevant scientific data must be acquired [24, 25]. Therefore, this study can provide more physiological information about IA and assists other researchers in realizing the regulation of the ANS. The results will benefit the further application, early detection, therapy, and even early prevention.

## Methods

### Participants

The main participants in this study were college students. The experiment recruitment information was posted in the department billboard. College students who are over 20 years old without agoraphobia and emotional disorder can contact the experimenter and participate in the experiment. Thirty-four college students between the ages of 21 and 27 (*μ* = 21.97, *SD* = 2.04), comprising 6 females and 28 males from the computer science department, participated in this experiment. Participants were classified into a HIA group (*n* = 19) and a LIA group (*n* = 15) using the Chen internet addiction scale (CIAS) with a cut-off score of 63 [26]. This investigation was approved by the Institution Review Board of the National Taiwan University Hospital, Hsinchu Branch (Hsinchu, Taiwan), under research project no.100IRB-32.

### Material and data collection

An emotional induction experiment was performed. CIAS, emotional induction materials, physiological signal acquisition equipment, and questionnaires for psychological and personal information were utilized. CIAS developed by Chen et al. is the main classification method of IA in this study. CIAS consists of 26 items, each item scaled by four points, and is a self-assessment scale including five dimensions of compulsive use, withdrawal, tolerance, problems in interpersonal relationship, and health/time management. The total CIAS scores range from 26 to 104, and cut-off points consolidated by psychiatrists’ diagnostic interview is reliable. The cut-off point for diagnosis is 63/64 [26, 27], and people with CIAS scores over 63 are regarded as high-risk IA (HIA) abusers. Five emotional films were selected as emotion-inducing materials from the database of Taiwan corpora of Chinese emotions and relevant psychophysiological data. The emotional content, valence and arousal levels of five film clips were anger (valence: 2.13 ± 1.23, arousal: 6.25 ± 2.2), fear (valence: 3.56 ± 1.82, arousal: 6.11 ± 2.09), sadness (valence: 3.35 ± 1.36, arousal: 4.63 ± 2.13), happiness (valence: 7.33 ± 1.38, arousal: 5.03 ± 2.34), and surprise (valence: 6.63 ± 1.60, arousal: 4.56 ± 2.26), respectively [28]. According to dimensional models of emotion [29], anger, sadness, and fear are negative emotions, while happiness and surprise are positive emotions.

Electrocardiography (ECG, with three disposable pregelled Ag/AgCl spot electrodes) was used with electrodes applied to the surface of a participant’s skin (right subclavian, left subclavian and lowest left rib). ECG signals were sampled at 1000 Hz and acquired using the DAQcard (USB 6218, National Instruments Corp., Austin, USA). Respiratory signals were collected using two respiratory belts (SS5LB, BIOPAC Systems, Inc., Goleta, USA) at a sampling rate of 1000 Hz. Facial images were captured using a webcam [Logitech, V-UBK45, USB 2.0, 10 fps (640 × 480), CA]. The ECG signal was the target signal and the respiratory signal and facial image were used only to elucidate the mechanism of ANS regulation.

### Experimental procedure

Participants were seated in a comfortable chair and signed an informed consent form when they understood the content and purpose of the experiment. The participants were asked to complete a CIAS questionnaire and to provide personal basic information. Before their emotions were induced using the film clips, electrodes were attached and ECG signals were checked. The resting ECG baseline (180 s) was measured before any emotion was induced. During the period of emotional induction, five emotional clips (three negative and two positive) were randomly displayed, each lasting 120–180 s. Participants completed a questionnaire to rank the intensity of their emotion (0–8) after seeing each clip. Before and after each clip was played, the participant performed a distracting task for 60 s and looked at a gray screen for 180 s to calm their emotion [28]. The total experiment time was 75 min. RSA data acquired during the gray screen displayed for 180 s is the stage of before emotional induction, and the during recording of the emotional induction was the stage of after emotional induction.

### Data analysis

The descriptive statistics and inferential statistics were both adopted [30]. The demographic information, personal basic physiological information, and experimental environment of HIA and LIA groups were examined using the Mann–Whitney U test and Chi square test. A *p* value less than 0.05 indicated statistical significance. The RSA values of negative and positive emotions in HIA and LIA groups were examined by Mann–Whitney U test, paired t-test, factorial ANOVA, and Holm–Bonferroni for multiple comparisons. The statistical calculations were performed using IBM SPSS Statistics 22 software (IBM, New York, USA).

**Fig. 1:**
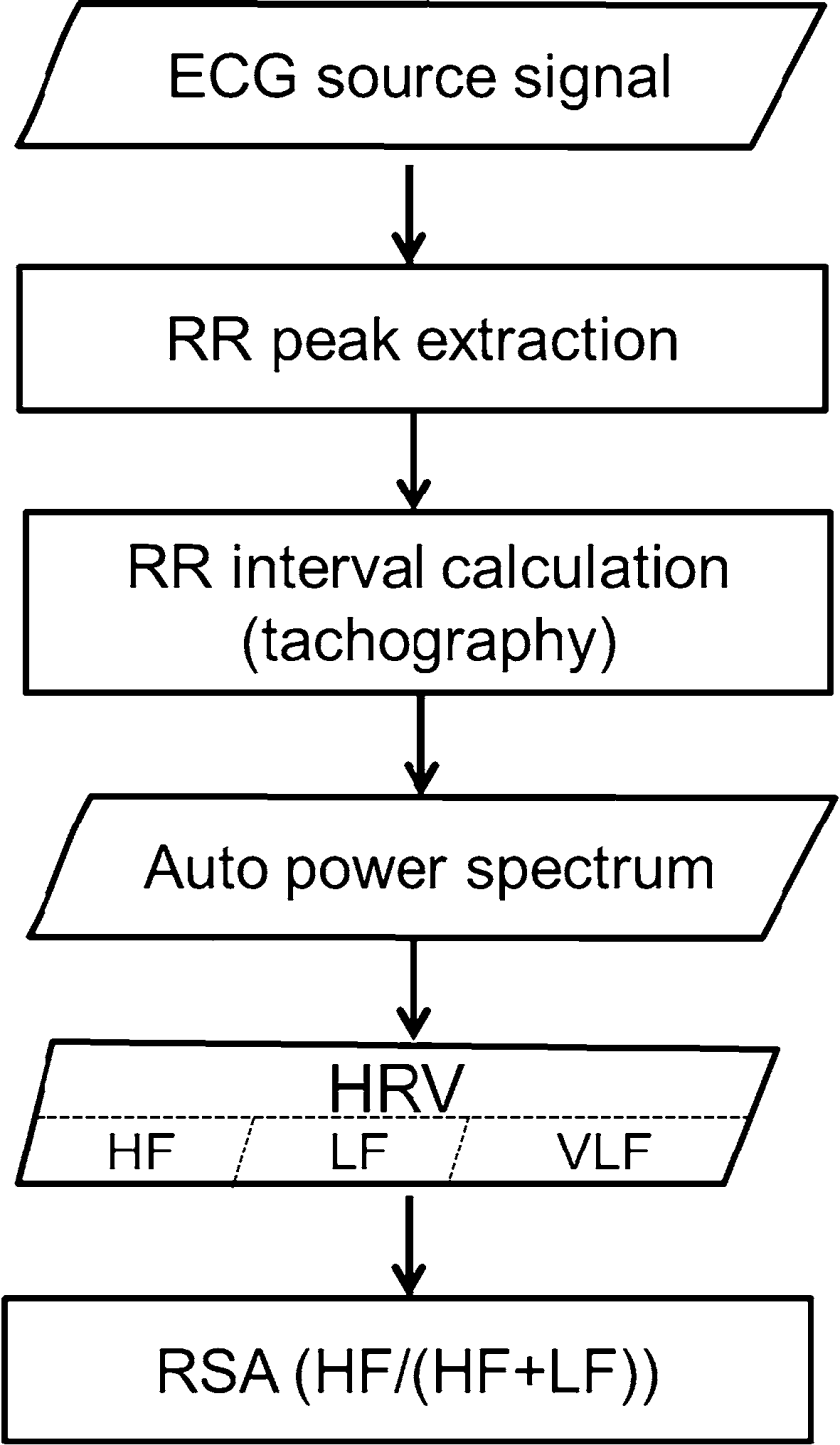
The analytical procedure of RSA

**Fig. 2:**
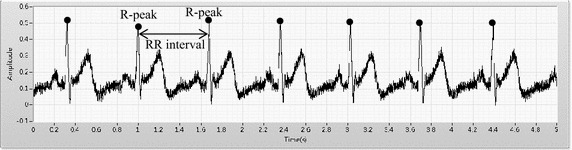
The ECG original signal (one HIA participant). The *black point* indicates R-peak, and the difference between R-peaks is RR interval

A program that was developed by the authors using LabVIEW 2012 (National Instruments, Austin, USA) was used to analyze the ECG signal, following a procedure that was presented in Fig. 1, and the ECG original data was shown in Fig. 2.

The ECG source signals were input to the program without filtering; one researcher then looked for R-waves peaks and another verified the accuracy of the findings of R peaks. The R–R peak interval was calculated and a tachograph was plotted [31]. The number of breathing cycles taken during the 120 and 180 s clips were 24–40 and 36–60, respectively. The RR intervals were transformed into an auto power spectrum, and HRV contains three frequency bands—high frequency (HF, 0.15–0.4 Hz), low frequency (LF, 0.04–0.15 Hz), and very low frequency (VLF, <0.04 Hz). Usually, HF indicates a parasympathetic nervous response and LF indicates a sympathetic nervous response. The RSA value was calculated as HF/(HF+LF). Five RSA-related emotional indices were tested; they were (1) the correlation coefficient between RSA value upon the emotional induction and the CIAS scores, (2) the significance of the relationship between RSA value and the five specified negative and positive emotions, (3) the difference between RSA value before and that after an emotion was induced (∆RSA), (4) the significance (*p* value) of ΔRSA between HIA and LIA, using Mann–Whitney U test. Finally, ∆RSA and CIAS scores for predicting IA were examined by linear model and multiple liner regression.

## Results

### Demographic and experimental environment information

The demographic information did not differ statistically significantly between HIA and LIA in gender (17 males, 2 females, and 11 males, 4 females, respectively, *p* = 0.22), age (*μ*_*HIA*_ = 21.90, *SE*_*HIA*_ = 0.49; *μ*_*LIA*_ = 22.07, *SE*_*LIA*_ = 0.52, *p* = 0.78), number of years of computer use (*μ*_*HIA*_ = 11.95 year, *SD*_*HIA*_ = 3.61; *μ*_*LIA*_ = 12.67 year, *SD*_*LIA*_ = 1.99, *p* = 0.41), or number of years of Internet use (*μ*_*HIA*_ = 10.21 year, *SD*_*HIA*_ = 3.10; *μ*_*LIA*_ = 11.33 year, *SD*_*LIA*_ = 2.77, *p* = 0.2). CIAS scores differed statistically significantly between HIA and LIA (*μ*_*HIA*_ = 69.37, *SE*_*HIA*_ = 1.32; *μ*_*LIA*_ = 55.87, *SE*_*LIA*_ = 1.07, *p* < 0.05). Since the environment may influence ANS activity, the CO_2_ concentration and room temperature were recorded; these two environmental parameters did not statistically significantly differ between HIA and LIA (CO_2_: *p* = 0.22, temperature: *p* = 0.57). The responses to the emotion questionnaire revealed that HIA reported weaker emotions than LIA, and the ranking scores for anger, fear, happiness, surprise in HIA and LIA groups were 6.58 ± 2.25, 7.01 ± 1.97, 6.95 ± 2.54, 6.55 ± 2.19, and 7.91 ± 0.33, 7.89 ± 0.40, 7.47 ± 2.00, 7.07 ± 2.17, respectively. But sadness was an exception (7.44 ± 1.85 and 6.93 ± 2.72 for HIA and LIA groups, respectively).So the subjective emotional intensity of HIA was inferred to be weaker than that of LIA [32].

### RSA-related indices

#### Statistical results and statistical test

**Fig. 3:**
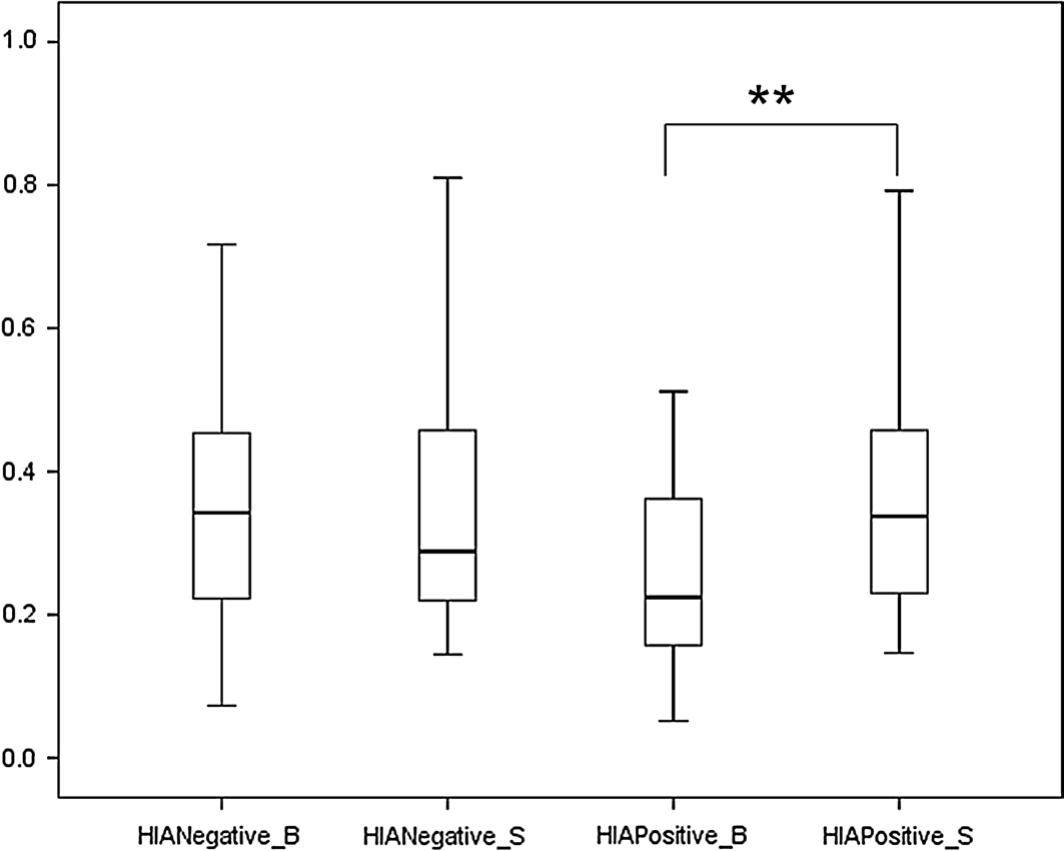
*Box plot* of RSA values of before and after emotional induction in the HIA group. HIANegative_B = RSA values of before negative emotional induction in the HIA group. HIANegative_S = RSA values of after negative emotional induction in the HIA group. HIAPositive_B = RSA values of before positive emotional induction in the HIA group. HIAPositive_S = RSA values of after positive emotional induction in the HIA group. ** is statistically significant at the 0.01 level (*p* < 0.01) using paired t-test

**Fig. 4:**
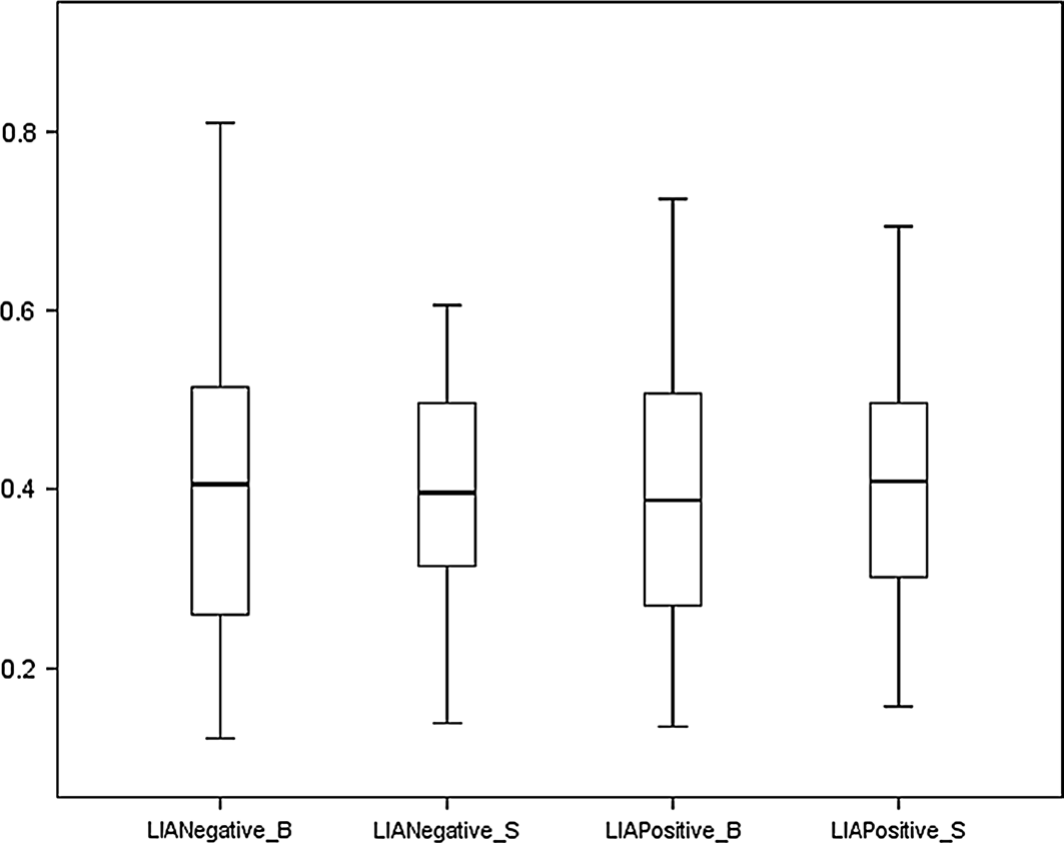
*Box plot* of RSA values of before and after emotional induction in the LIA group. LIANegative_B = RSA values of before negative emotional induction in the LIA group. LIANegative_S = RSA values of after negative emotional induction in the LIA group. LIAPositive_B = RSA values of before positive emotional induction in the LIA group. LIAPositive_S = RSA values of after positive emotional induction in the LIA group

Figures 3 and  4 and Tables 1 and 2 presented descriptive statistics and statistical tests of the RSA values upon the emotional induction of HIA and LIA groups.

**Table 1:** The descriptive statistics, normality test, paired t-test, and Mann–Whitney U test of RSA value upon the emotional inductions in the HIA group

Emotion		N	Mean	SD	Var	*P*_1_	*P*_2_	*P*_3_	Power (effect size)
Negative	B	57	0.33	0.16	0.03	0.20	0.27		0.21(0.16)
	S	57	0.36	0.15	0.02	0.20			
Anger	B	19	0.37	0.19	0.04	0.50	0.56	0.418	
	S	19	0.34	0.16	0.03	0.05*			
Fear	B	19	0.29	0.11	0.01	0.73	0.002**	0.007**	
	S	19	0.39	0.10	0.01	0.10			
Sadness	B	19	0.33	0.17	0.03	0.07	0.72	0.62	
	S	19	0.35	0.16	0.03	0.01*			
Positive	B	38	0.25	0.12	0.02	0.08	0.000**		0.76(0.99)
	S	38	0.37	0.16	0.03	0.03*			
Happiness	B	19	0.25	0.11	0.01	0.28	0.02*	0.04*	
	S	19	0.37	0.19	0.04	0.05			
Surprise	B	19	0.25	0.14	0.02	0.12	0.001**	0.01*	
	S	19	0.37	0.13	0.02	0.47			

*N* sample size number, *SD* standard deviation, *Var* variance, *HIA* high-risk internet addiction group, *N of negative* *57* (19 HIA*3 negative emotion), *N of positive* 38 (19 HIA*2 positive emotion), *N* *19* HIA people, *B* baseline (before emotional induction), *S* stimulation (after emotional induction), *P*_*1*_ normality test, *P*_*2*_ paired t test, *P*_*3*_ Mann–Whitney U test

* Statistically significant at the 0.05 level (*p* < 0.05)

** Statistically significant at the 0.01 level

**Table 2:** The descriptive statistics, normality test, paired t-test, and Mann–Whitney U test of RSA value upon the emotional inductions in the LIA group

Emotion		N	Mean	SD	Var	*P*_1_	*P*_2_	*P*_3_	Power (effect size)
Negative	B	45	0.405	0.17	0.03	0.62	0.98		0.05 (0.01)
	S	45	0.406	0.13	0.02	0.28			
Anger	B	15	0.39	0.14	0.02	0.39	0.86	0.83	
	S	15	0.38	0.14	0.02	0.69			
Fear	B	15	0.42	0.19	0.04	0.95	0.94	0.87	
	S	15	0.42	0.15	0.02	0.68			
Sadness	B	15	0.40	0.19	0.04	0.77	0.72	0.54	
	S	15	0.42	0.12	0.02	0.81			
Positive	B	30	0.399	0.17	0.03	0.26	0.70		0.07 (0.08)
	S	30	0.416	0.15	0.02	0.32			
Happiness	B	15	0.39	0.18	0.03	0.90	0.10	0.29	
	S	15	0.47	0.15	0.02	0.50			
Surprise	B	15	0.41	0.18	0.03	0.15	0.45	0.72	
	S	15	0.36	0.14	0.02	0.35			

*N* sample size number, *SD* standard deviation, *Var* variance, *LIA* low-risk internet addiction group, *N of negative* *45* (15 LIA*3 negative emotion), *N of positive* 30 (15 LIA*2 positive emotion), *N* *15* LIA people, *B* baseline (before emotional induction), *S* stimulation (after emotional induction), *P*_*1*_ normality test, *P*_*2*_ paired t-test, *P*_*3*_ Mann–Whitney U test

* Statistically significant at the 0.05 level (*p* < 0.05)

** Statistically significant at the 0.01 level

The RSA values were first analyzed by nonparametric statistics regardless of the sample size or distribution of the variables. After test the normality using the Shapiro–Wilk test (sample numbers <50) and the Kolmogorov–Smirnov test (sample numbers >50), almost the distribution of RSA values in the HIA and LIA groups were normal. Hence, the statistical power and effect size of RSA values upon the induction of negative and positive emotions were estimated for HIA and LIA groups using a well-known application software, G*power 3.1. Power ($1 - \beta$) and effect size (Cohen’s d) of RSA value upon before and after the induction of negative emotion in the HIA group were 0.21 and 0.16, respectively; that of the positive emotion were 0.76 and 0.99, respectively. Power and effect size of RSA value upon before and after the induction of negative emotion in the LIA group were 0.05 and 0.01, respectively; that of the positive emotion were 0.07 and 0.08, respectively. Power of RSA value upon the induction of negative emotion of the HIA and LIA groups was 0.36; that of the positive emotion was 0.21. The power value and effect size upon before and after the induction of positive emotion in the HIA group were high (0.76) and large (0.99). Although most powers and effect sizes of RSA value of emotions were not high, fear, happiness, surprise differed statistically significantly in the HIA group, and these differences were large enough to be detected. The low power and small effect size may be caused by small sample size and the RSA values upon the emotional inductions in the HIA group were not as expected or known in advance, and so the power might not have been determined accurately.

Second, the RSA values upon the before and after induction of negative and positive emotions in the HIA group and in the LIA group were examined using Mann–Whitney U test (small and unequal sample size of two groups) and paired t-test, respectively. The RSA values of positive emotion before and after induction differed statistically significantly in the HIA group (*p* < 0.00). Third, RSA values upon the induction of five emotions were examined using Mann–Whitney U test, paired t-test (specific emotion before and after emotional induction), and factorial ANOVA. In the HIA group, the RSA values upon before and after the induction of fear, happiness, surprise statistically significantly differed using whether Mann–Whitney U test or paired t-test. For the LIA group, the RSA values of five emotions did not present statistically difference. The RSA values of the HIA group and the LIA group upon before or after five emotional inductions were also tested, and the result was shown in Table 3. The factorial ANOVA was conducted to test the effect among IA groups (HIA, LIA), emotional types (five emotions), and emotional induction states (before or after emotional inductions).

**Table 3:** Factorial analysis of variance of RSA value upon 2 IA groups (HIA, LIA) five emotions (anger, fear, sadness, happiness, surprise) and 2 emotional induction states (before positive emotional induction, after negative emotional induction)

Variables	SS	df	MS	F	P
IA	0.21	1	0.21	16.94	0.000**
Emotion	0.04	4	0.01	0.82	0.52
Induction state	0.03	1	0.03	2.64	0.11
IA* emotion	0.06	4	0.02	1.18	0.32
IA* induction state	0.21	1	0.21	16.94	0.000**
Emotion* induction state	0.04	4	0.01	0.82	0.52
IA* emotion* induction state	0.06	4	0.02	1.18	0.32
Error	3.69				
Total	39.50				

*IA* internet addiction groups (HIA, LIA)

* Statistically significant at the 0.05 level (*p* < 0.05)

** Statistically significant at the 0.01 level (*p* < 0.01)

The interaction among IA groups, emotional types and emotional induction states was not statistically significant (*p* = 0.32). The interaction between IA groups and emotional induction states was statistically significant (*p* = 0.00 <0.01). The HIA group and the LIA group statistically significantly differed (*p* = 0.00 <0.01). But it was no any statistically significant difference between emotion types or emotional induction states.

**Fig. 5:**
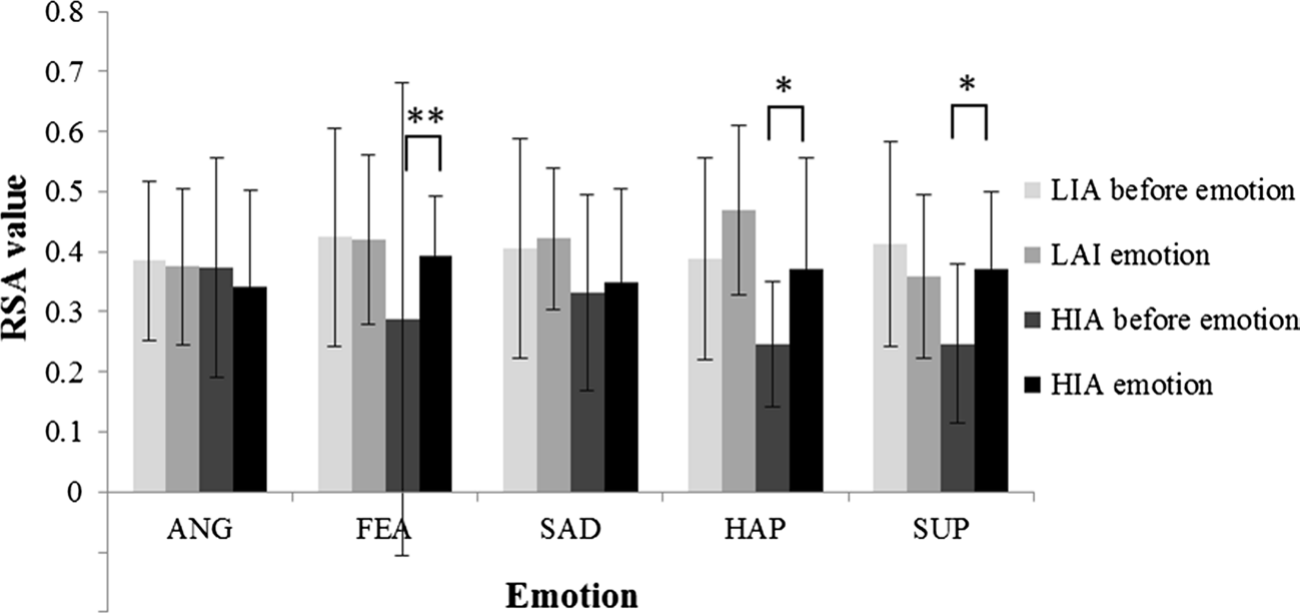
RSA values upon before and after emotional induction of HIA and LIA groups. *ANG* anger, *FEA* fear, *SAD* sadness, *HAP* happiness, *SUP* surprise, ** and * are statistically significant (*p* < 0.01 and *p* < 0.05, respectively)

Figure 5 presented the RSA values upon before and after emotional induction of HIA and LIA groups.

The RSA values of HIA were lower than those of LIA both before and after the induction of positive and negative emotions. When participants experienced a negative emotion (anger or fear), excluding sadness, their RSA values declined; the decline for HIA was greater than that for LIA. When participants experienced a positive emotion (happiness or surprise), their RSA values increased, with the exception that the RSA values of LIA participants who experienced surprise decreased. The RSA values of HIA participants before induction of fear, happiness, or surprise statistically significantly differed from that after induction of those emotions, with *p* values of 0.007, 0.04 and 0.01, respectively.

The changes in RSA values upon the emotional induction were denoted as ΔRSA (RSA value after emotional induction minus RSA value before emotional induction). The *p* value for the difference between the ΔRSA values of HIA and LIA for anger, fear, sadness, and happiness were 0.7, 0.24, 0.073 and 0.69, respectively, whereas that for surprise was 0.03 (statistically significant).

### Correlation coefficient

Tables 4, 5 and 6 presented the correlation coefficient between the RSA values and the CIAS scores in different emotional states for all participants, the HIA and LIA groups, respectively. Paired t-test was used to examine the statistically significant correlation among those emotions, and then the *p* values were corrected using Holm–Bonferroni for multiple comparisons.

**Table 4:** The correlation coefficient between RSA value upon the emotional induction and the CIAS scores for all participants

Emotion (#)		Negative (102)		Positive (68)		ANG (34)		FEA (34)		SAD (34)		HAP (34)		SUP (34)		CIAS (34)
		B	S	B	S	B	S	B	S	B	S	B	S	B	S	
Negative (102)	B	1														
	S	0.22*	1													
Positive (68)	B			1												
	S			0.25*	1											
ANG (34)	B					1										
	S					0.15	1									
FEA (34)	B					−0.01	0.40*	1								
	S					0.23	0.57**	0.19	1							
SAD (34)	B					0.04	0.03	0.05	0.21	1						
	S					−0.09	0.36	0.23	0.59**	0.36*	1					
HAP (34)	B					0.25	0.31	0.49**	0.28	0.05	0.04	1				
	S					0.23	0.70**	0.37*	0.52**	0.01	0.46*	0.38*	1			
SUP (34)	B					0.16	0.16	0.10	0.10	0.24	0.29	0.06	0.26	1		
	S					0.43	0.58**	0.19	0.78**	0.09	0.50**	0.24	0.56**	0.13	1	
CIAS (34)						0.07	−0.18	−0.46**	−0.19	−0.22	−0.41**	−0.32	−0.31	−0.40*	−0.20	1

*p* values were corrected using Holm–Bonferroni for multiple comparisons, and no any statistically significantly differed

*B* baseline (before emotional induction), *S* stimulation (after emotional induction); *AN* anger, *FEA* fear, *SAD* sadness, *HAP* happiness, *SUP* surprise, *CIAS* Chen internet addiction scale, *#* sample size number

* Correlation is statistically significant at the 0.05 level (2-tailed) using paired t-test

** Correlation is statistically significant at the 0.01 level (2-tailed) using paired t-test

**Table 5:** The correlation coefficient between RSA value upon the emotional induction and the CIAS scores in the HIA group

Emotion (#)		Negative (57)		Positive (38)		ANG (19)		FEA (19)		SAD (19)		HAP (19)		SUP (19)		CIAS (19)
		B	S	B	S	B	S	B	S	B	S	B	S	B	S	
Negative (57)	B	1														
	S	0.20	1													
Positive (38)	B			1												
	S			0.32	1											
ANG (19)	B					1										
	S					0.21	1									
FEA (19)	B					0.05	0.46	1								
	S					0.10	0.67	0.34	1							
SAD (19)	B					0.05	−0.45	−0.20	0.00	1						
	S					−0.19	0.47	0.12	0.62	0.24	1					
HAP (19)	B					0.32	0.23	0.44	0.07	0.10	−0.28	1				
	S					0.33	0.77**	0.35	0.56	−0.11	0.37	0.19	1			
SUP (19)	B					0.30	0.61**	0.23	0.41	0.31	0.44	0.02	0.57*	1		
	S					0.43	0.71**	0.37	0.69	0.08	0.49*	0.00	0.62**	0.50*	1	
CIAS (19)						0.14	−0.31	−0.20	−0.17	0.07	−0.26	0.24	−0.16	−0.25	−0.50	1

*p* values were corrected using Holm–Bonferroni for multiple comparisons, and no any statistically significantly differed

*B* baseline (before emotional induction), *S* stimulation (after emotional induction); *AN* anger, *FEA* fear, *SAD* sadness, *HAP* happiness, *SUP* surprise, *HIA* high-risk internet addiction group, *CIAS* Chen internet addiction scale, *#* sample size number

* Correlation is statistically significant at the 0.05 level (2-tailed) using paired t-test

** Correlation is statistically significant at the 0.01 level (2-tailed) using paired t-test

**Table 6:** The correlation coefficient between RSA value upon the emotional induction and the CIAS scores in the LIA group

Emotion (#)		Negative (45)		Positive (30)		ANG (15)		FEA (15)		SAD (15)		HAP (15)		SUP (15)		CIAS (15)
		B	S	B	S	B	S	B	S	B	S	B	S	B	S	
Negative (45)	B	1														
	S	0.19	1													
Positive (30)	B			1												
	S			0.11	1											
ANG (15)	B					1										
	S					−0.03	1									
FEA (15)	B					−0.18	0.39	1								
	S					0.38	0.49	0.07	1							
SAD (15)	B					−0.03	0.11	0.10	0.41	1						
	S					0.18	0.07	0.27	0.64*	0.48	1					
HAP (15)	B					0.25	0.39	0.34	0.37	−0.15	0.20	1				
	S					−0.02	0.53	0.28	0.53	0.10	0.63*	0.44	1			
SUP (15)	B					−0.02	−0.51	−0.40	−0.16	0.06	−0.08	−0.34	−0.38	1		
	S					0.45	0.40	0.10	0.86**	0.12	0.74**	0.54	0.57*	−0.17	1	
CIAS (15)						0.24	0.19	−0.26	−0.21	−0.33	−0.26	−0.06	−0.15	−0.21	0.28	1

*p* values were corrected using Holm–Bonferroni for multiple comparisons, and no any statistically significantly differed

*B* baseline (before emotional induction), *S* stimulation (after emotional induction); *AN* anger, *FEA* fear, *SAD* sadness, *HAP* happiness, *SUP* surprise, *LIA* low-risk internet addiction group, *CIAS* Chen internet addiction scale, *#* *#* sample size number

* Correlation is statistically significant at the 0.05 level (2-tailed) using paired t-test

** Correlation is statistically significant at the 0.01 level (2-tailed) using paired t-test

For all participants, the RSA values before emotions were induced were moderately or weakly correlated with that after the emotion was induced. All the corrected *p* values were no statistically significant correlation.

In each of the HIA and LIA groups, the RSA values before a negative or positive emotion was induced were weakly correlated with that after the emotion was induced. Moreover, in both groups, RSA value in one specific emotion state was almost moderately correlated with any other emotion. In the HIA group, the RSA value before a surprise emotion was induced was statistically significantly correlated with that afterward (*r* = 0.5). Anger was positively and statistically significantly correlated with happiness and surprise. Sadness and happiness were positively and statistically significantly correlated with surprise. But all above corrected *p* values using Holm–Bonferroni showed no statistically significant correlation. In the LIA group, fear was positively and statistically significantly correlated with sadness and surprise. Sadness was positively and statistically significantly correlated with happiness and surprise, and happiness was positively and statistically significantly correlated with surprise. But all above corrected *p* values using Holm–Bonferroni showed no statistically significant correlation. Most correlations between RSA values and CIAS scores in both groups were moderately. It is interesting that in the HIA group, the anger and surprise was negatively correlated, whereas in the LIA group was positively correlated.

### Linear model

Finally, a linear model was established by ∆RSA and CIAS scores for predicting IA, yielding the results in Table 7.

**Table 7:** Linear model, multiple linear regressions of CIAS score and changes in RSA upon the emotional induction

Emotion	Anger		Fear		Sadness		Happiness		Surprise	
	$\left| {\Delta {\text{RSA}}} \right|$	$\Delta {\text{RSA}}$	$\left| {\Delta {\text{RSA}}} \right|$	$\Delta {\text{RSA}}$	$\left| {\Delta {\text{RSA}}} \right|$	$\Delta {\text{RSA}}$	$\left| {\Delta {\text{RSA}}} \right|$	$\left| {\Delta {\text{RSA}}} \right|$	$\left| {\Delta {\text{RSA}}} \right|$	$\left| {\Delta {\text{RSA}}} \right|$
All participants (#34)										
F	0.85	0.72								
p_1_	0.53	0.62								
t	0.38	−0.81	−1.64	1.11	0.08	0.05	−0.22	−0.20	−1.25	0.84
p_2_	0.71	0.43	0.12	0.28	0.94	0.96	0.83	0.84	0.26	0.41
HIA (#19)										
Slope	3.01	−9.14	6.40	1.53	1.68	−10.04	−11.24	−8.26	−22.03	−10.25
Residue	32.22	28.50	31.31	31.55	32.31	28.44	29.74	29.84	24.35	30.58
F	0.72	0.68								
p_1_	0.62	0.65								
t	0.72	−0.85	0.41	0.54	0.28	−0.33	−1.21	−0.53	−1.37	−0.82
p_2_	0.48	0.42	0.69	0.60	0.78	0.75	0.25	0.61	0.20	0.43
LIA (#15)										
Slope	−15.75	−0.95	−15.77	1.53	−33.21	3.14	4.18	−1.80	4.53	−5.86
Residue	14.71	18.03	13.79	17.01	8.94	17.76	16.98	17.03	17.64	16.14
F	2.23	0.30								
p_1_	0.16	0.90								
t	−1.96	0.43	−0.46	0.62	−2.23	0.47	0.25	−0.43	0.14	−1.08
p_2_	0.09	0.68	0.66	0.56	0.06	0.66	0.81	0.68	0.89	0.32

ΔRSA denotes the changes in RSA values before and after emotional induction (RSA value after emotional induction subtracting RSA value before emotional induction). |∆RSA| denotes the changes in RSA values before and after emotional induction without increment or decrement of RSA reactivity. F value and p_1_ values are the ANOVA result using multiple linear regression analysis for all 5 emotions. t value and p_2_ values are the coefficients results using multiple linear regression analysis

After emotions were induced, the RSA values were either positive (+) or negative (−), revealing the direction of RSA reactivity. Two $\Delta {\text{RSA}}$ values, the original $\Delta {\text{RSA}}$ and $\left| {\Delta {\text{RSA}}} \right|$ (which eliminates the direction of RSA reactivity), were considered. A slope of the linear model of greater than zero indicated that the relationship between $\Delta {\text{RSA}}$ or $\left| {\Delta {\text{RSA}}} \right|$ and the CIAS score was positive. In the HIA group, the most slope values for negative emotions exceeded zero so the $\left| {\Delta {\text{RSA}}} \right|$ value increased with the CIAS score, but for positive emotions, the $\Delta {\text{RSA}}$ values became lower as the CIAS score increased. In the LIA group, for negative emotions, as the CIAS score increased, the $\left| {\Delta {\text{RSA}}} \right|$ value decreased, but for positive emotions, as the CIAS score increased, the $\left| {\Delta {\text{RSA}}} \right|$ value increased. Therefore, HIA and LIA exhibited opposite RSA reactions to positive and negative emotions. Further, for all participants, the HIA and LIA groups, F value and p values for the model, together with the coefficients, t and *p* values for each emotion predictors using multiple linear regression analysis were shown in Table 7. Results indicated that difference of RSA values in five emotional states were not strong predictors and the reasons might be less participant number or emotion types.

## Discussion

The relationship between the RSA value and five emotional inductions of HIA and of LIA was investigated. We hypothesized that IA affects the reactivity of RSA to emotions. According to the relevant literature, people with a higher resting RSA level express more positive emotions and suppress the expression of negative emotions [22, 23]. HIA people can be regarded as users with high risk addicts, while LIA people can be regarded as low risk addicts. From the results herein, the RSA reactivity of HIA to both positive and negative emotions is lower than that of LIA, implying that the HIA group tended to express negative emotions rather than positive emotions, and did not suppress negative emotion. The subjective emotional assessment revealed that HIA exhibited a lower emotional intensity than LIA, except with regard to sadness. Hence, the subjective emotional intensity of people with IA may be lower than that of non-IA people, and herein, such people exhibited a weaker objective RSA reactivity to emotions, and they preferred to express negative emotions.

Based on the literature [22], with respect to negative emotions, the RSA reactivity (RSA value) of the two groups was expected to decrease following the induction of negative emotion, and the results concerning RSA reactivity were consistent with the literature in this respect. Specifically, the RSA reactivity of fear statistically significantly decreased of HIA. However, the RSA reactivity of sadness increased in both HIA and LIA groups. Furthermore, since HIA may prefer to express negative emotions over positive ones, the RSA reactivity of negative emotions of HIA should be lower than that of LIA. The RSA reactivity following the induction of fear confirms this expectation.

For positive emotion, RSA reactivity increased or more activated, and the parasympathetic reactivity is stronger. The RSA reactivity of a positive emotion of the two groups was increased and this finding is consistent with the literature [23]. The RSA reactivity of HIA biologically significantly increased. However, the RSA reactivity of LIA decreased when surprise was induced. HIA expressed less positive emotion than LIA. The RSA reactivity following the induction of positive emotions of HIA was expected to be lower than that of LIA, and the results confirmed this expectation except with respect to surprise.

RSA reactivity mainly reflects the regulation by the vagus nerve (parasympathetic activity). Cardiac vagal control indicates self-regulatory tendencies and spontaneous self-regulation is related with resting RSA level for individuals [22], and common neural pathways existed in underlying autonomic control and self-regulation [19, 21]. HIA exhibited lower parasympathetic activity when an emotion was induced. To determine the relationship between tendency toward IA and RSA reactivity, a linear model of the relationship between CIAS scores and change in RSA values was established. In the HIA group, the CIAS score was positively related to change in RSA value in response to negative emotions, but negatively correlated in response to positive emotions, suggesting that people with higher CIAS scores exhibited stronger regulation of RSA reactivity following negative emotion, but the regulation of RSA reactivity following positive emotion was low. For the LIA group, unlike the HIA group, the CIAS score and change in RSA value were negatively correlated in response to negative emotions, but positively correlated in response to positive emotions, suggesting that people without IA who experience negative emotions exhibited weaker regulation of RSA reactivity than people with IA but those who experience positive emotion exhibited a strong regulating effect. The mechanism of regulation warrants investigation.

## Limitations

First, the sample size was not large and the power and effect size were not high. More sample sizes should be recruited in the future study, and the results of the relationship between RSA and IA will be more robust and consolidated. Second, the play sequence of five emotional film clips in main experiment procedure was random. This random sequence is designed to approach real world condition. However, the total number of sequences could be 120, but the 34 sample size cannot fit for all 120 sequences, and the sample size will expected very large. This study may provide an opportunity to find out meaningful play sequences, and the less sequence can reduce the variation in study. Following the restricted play sequence, the estimated sample size would be smaller than that with 120 play sequences, and this benefits experiment conduct, and the reliability of experiment result. Third, the purpose of using internet was not limited, so the types of internet addiction were varied. Among many purposes of using internet, we will focus on internet gaming disorder (IGD). Because online game player is a big population of IA abusers, and many study results were reported; furthermore, it probably will be included in behavioral addiction by DSM in the future. The RSA reactivity or autonomic nervous responses for IGD abusers were expected to be further investigated.

## Conclusions

An experiment in which emotions were induced was conducted to acquire psychological and physiological information, including respiratory signals, ECG signals, facial images, and self-assessed emotional intensity, from participants who experienced those emotions. RSA value, computed from the ECG signals, was the main variable that reflected ANS activity, and especially vagus nerve regulation. RSA values and changes upon the induction of negative and positive emotions were examined in 19 HIA and 15 LIA participants.

The results thus revealed that the changes in RSA values biologically significantly differed between HIA and LIA, especially when sadness, happiness, or surprise was induced. The results demonstrate that HIA had a lower RSA level than LIA both before and after emotional induction, and that their emotional reactions (as captured by an RSA-related index) were consistent with the literature. However, the changes in RSA level upon the induction of surprise and sadness were not consistent with the literature. The regulation of the ANS may differ between people with IA and people without IA. Additionally, the relationship between the change in RSA values and CIAS score was modeled using a linear predictor and the two groups were found to exhibit opposite RSA reactivity in both negative and positive emotional states. People with higher CIAS scores (HIA) exhibited stronger RSA reactivity following negative emotion, but the RSA reactivity following positive emotion was weaker. Nevertheless, the post hoc test of RSA values of emotions did not show much statistically significant difference. The number of participants should increase to improve the statistical power. This investigation provides results concerning the RSA reactivity, the vagus nerve activity, of IA and assists further study of the regulation of the ANS for IA abusers. The results will benefit the further application, early detection, therapy, and even early prevention.

## Abbreviations

ANS: autonomic nervous system
ANOVA: analysis of variance
CIAS: Chen internet addiction scale
DSM: diagnostic and statistical manual for mental disorders
ECG: electrocardiography
HRV: heart rate variability
HF: high frequency
HIA: high-risk internet addiction
IA: internet addiction
IGD: internet gaming disorder
LIA: low-risk internet addiction
LF: low frequency
RSA: respiratory sinus arrhythmia
VLF: very low frequency
